# Higher Frequency of NK and CD4^+^ T-Cells in Mucosa and Potent Cytotoxic Response in HIV Controllers

**DOI:** 10.1371/journal.pone.0136292

**Published:** 2015-08-20

**Authors:** Natalia Andrea Taborda, Sandra Milena González, Cristiam Mauricio Alvarez, Luis Alfonso Correa, Carlos Julio Montoya, María Teresa Rugeles

**Affiliations:** 1 Grupo Inmunovirología, Facultad de Medicina, Universidad de Antioquia, UdeA, Medellín, Colombia; 2 Grupo de Inmunología Celular e Inmunogenética, Facultad de Medicina, Universidad de Antioquia, UdeA, Medellín, Colombia; 3 Sección de Dermatología, Departamento de Medicina interna, Facultad de Medicina, Universidad de Antioquia, UdeA, Medellín, Colombia; 4 Coordinador Laboratorio de Patología, Laboratorio Clínico VID, Obra de la Congregación Mariana, Medellín, Colombia; Harvard Medical School, UNITED STATES

## Abstract

HIV infection induces immune alterations, mainly in gut mucosa, where the main target cells reside. However, the evolution of the infection is variable among infected individuals, as evidenced by HIV controllers who exhibit low or undetectable viral load in the absence of treatment. The aim of this study was to evaluate the frequency, phenotype and activity of T and NK cells in peripheral blood and gut mucosa in a cohort of Colombian HIV controllers. Blood and gut biopsies were included. The frequency and the activation status of T and NK cells were performed by flow cytometry. In addition, Gag-stimulated CD8^+^ T-cells and cytokine-stimulated NK cells were tested for cytotoxic activity. Finally, microbial translocation was measured by plasma lipopolysaccharide quantification. Compared with HIV-progressors, HIV controllers exhibited higher frequency of CD4^+^ T and NK cells, and lower expression of activation molecules in blood and mucosal immune cells, as well as lower microbial translocation. An increased production of molecules associated with cytotoxic activity of CD8^+^ T-cells in blood and mucosa and a higher percentage of polyfunctional CD8^+^ T cells in blood were also observed in HIV controllers. In addition, an increased activity of NK cells was observed in blood. These findings suggest that HIV controllers have a potent immune response, mainly mediated by cytotoxic cells that control HIV replication, which contribute to reducing alterations at the gut mucosa.

## Introduction

One of the most important pathogenic mechanisms during HIV infection is immune hyperactivation [1], which is induced initially by viral antigens, and then exacerbated by the destruction of the gut-associated lymphoid tissue (GALT), the major site for HIV replication [2]. Viral replication leads to the loss of mucosal integrity and translocation of microbial products, such as lipopolysaccharides (LPS), from intestinal lumen to systemic circulation [3]. As a result, there is an increased number of activated cells, apoptosis, mainly of CD4^+^ T-cells, anergy, and a generalized immune dysfunction [2]. Compiled evidence suggests that the immune response achieved in GALT could be the key factor to control viral replication, delaying AIDS progression [4].

The natural history of HIV infection is heterogeneous [5]. There are HIV controllers, who exhibit a spontaneous and sustained control of viral replication, at least for one year, without antiretroviral therapy (HAART) [6]. Although mechanisms related to the viral control are not completely understood, the cytotoxic response seems to play an important role in delaying AIDS progression [7,8].

NK cells are widely distributed in the body, including the gastrointestinal tract and peripheral blood (PB) [9]. Depending on the expression of CD16 and CD56 molecules, these cells can be classified in the following subsets: i) CD56^dim^ (CD16^+^ or CD16^-^) with a high cytotoxic response; ii) CD56^bright^, mostly cytokine producers; and iii) CD56^-^, that exhibits functional alterations and accumulate in patients with advanced AIDS [10]. In intestinal mucosa, NK cells are located mainly in intraepithelial compartments and lamina propria [9]. Previous reports showed an increased frequency of NK cells in GALT as viral load decreases after HAART [11]. In addition, the frequency of intraepithelial NK cells has been positively correlated with CD4^+^ T-cell counts [12].

Additionally, cytotoxic T-lymphocytes (CTLs) have also been associated with viral control [7,8,13]. Long-term-non progressors (LTNP) exhibit polyfunctional CTL responses in PB and GALT [7,14].

Based on this, we hypothesized that compared to HIV progressors, HIV controllers have increased proportions of adaptive and innate cytotoxic cells with enhanced functionality in the PB and the GALT, which might contribute to preserve the integrity of the gut mucosa. To test this, we evaluated the frequency of T and NK cell subpopulations, their activation level, and their function in PB and GALT, as well as the level of microbial translocation in a cohort of Colombian HIV controllers who were compared with HIV-progressors.

## Material and Methods

### Study Population

Two groups of HIV-infected individuals recruited from health insurance programs in Medellín-Colombia were included: i) 14 HIV controllers, defined as previously described [15]; briefly, they are patients who have been infected for more than one year, naïve for antiretroviral therapy, and exhibiting a spontaneous and sustained control of viral replication with viral loads lower than 2000 copies/mL; and ii) 18 chronic HIV-progressors, who exhibited CD4^+^ T-cell counts >250 cells/μL, HIV viral load between 10.000–100.000 RNA copies/mL and naïve for antiretroviral therapy (**Table 1**). Gut biopsies from 10 HIV controllers and 12 progressors from these two cohorts were available.

**Table 1:** Demographic and clinical information.

	HIV controllers(n = 14)	Progressors (n = 18)	*p* value
**Age in years,Median (range)**	28(20–49)	30 (19–50)	0.6480
**Gender,Male:Female**	8: 6	16: 2	N/A
**Time of diagnosis in months Median (range)**	49(12–168)	51 (12–120)	0.8941
**Plasma HIV-1 viral load in RNA copies/mL Median (range)**	211(20–1885)	31552 (11206–160405)	**<0.0001**
**CD4** ^**+**^ **T cell counts Median (range)**	745(514–1367)	443(267–819)	**0.0083**

N/A: Not applicable

Thirteen HIV controllers were homozygous for the wild-type CCR5 allele and one was heterozygous (CCR5-delta32), while all HIV-progressors were negative for the delta32 mutation. The individuals enrolled signed a written informed consent prepared according to the Colombian Legislation, Resolution 008430/1993; the present study and the inform consent were approved by the Ethical Committee (Comité de Bioética Sede Investigación Universitaria CBEIH-SIU) of Universidad de Antioquia (certificate 11-08-352).

Allelic frequencies of those HLA alleles previously associated with resistance to HIV were similar in HIV controllers and progressors.

### Viral load

Plasma viral load (VL) was determined using the commercial assay RT-PCR Ampliprep-Cobas (Roche, Indianapolis, IN, USA), following the manufacturer’s protocol, with a detection limit of 20 copies/mL.

### Isolation of peripheral blood and rectal cells

Peripheral blood mononuclear cells (PBMCs) were obtained by centrifugation on Histopaque (Sigma-Aldrich, St Louis, MO, USA) for 30 min at 400 *g*. Rectosigmoidoscopy was performed as previously reported [16]. Fifteen tissue samples were obtained and processed using 0.5 mg/mL collagenase type II from *Clostridium histolyticum* (Sigma-Aldrich) diluted in RPMI-1640 and 7.5% fetal bovine serum (FBS) plus 100 U/mL penicillin and 100 μg/mL streptomycin (Gibco-BRL, Grand Island, NY, USA), for 30 min at 37°C with shaking. Fragments were then disrupted by passage through a 30 mL syringe with a blunt end 16 gauge needle (Stem Cell Technologies, Vancouver, BC, Canada). A 70 mM nylon strainer (Falcon, Lincoln Park, NJ, USA) was used to isolate rectal cells (RCs). PBMCs and RCs were washed with Dulbeco’s PBS (DPBS) (Sigma-Aldrich). For all assays we used fresh cells. During the optimization of cell cultures we evaluated cell viability by staining with LIVE/DEAD Cell Viability Assays (Life Technologies) and we observed over 85% of viability.

### Antibodies

The following fluorochrome-labeled mouse monoclonal antibodies were from Becton Dickinson (BD Biosciences, San Jose, CA, USA): anti-CD8 (clone RPA-T8), IFN-γ (clone: 4S.B3), IL-2 (clone MQ1-17H12), MIP-1β (clone D21-1351) and granzyme B (clone GB11); and from eBioscience (San Diego, CA): anti- CD3 (clone UCHT1), CD16 (clone CB16), CD56 (clone CMSSB), HLA-DR (clone LN3), CD38 (clone HIT2), CD69 (clone FN50), TNF-α (clone MAb11) and CD4 (clone OKT-4). In addition, we used CD45 (clone J.33) from Beckman Coulter (Fullerton, CA, USA) and perforin (clone B-D48) from BioProducts (Middletown, MD, USA).

### Flow cytometry

In order to determine the frequency and phenotype of NK and T cells, 150 mL of whole blood were incubated for 30 min at 4°C with the following antibodies for NK cells: anti-CD3-Pacific blue, CD16-FITC, CD56-APC, CD69-PE and CD45 PECy7; and for T-cells: anti-CD3-APC-Cy7, CD4-APC, CD8-Pacific blue, HLA-DR-FITC and CD38-PECy7. Red blood cells were eliminated by lysis buffer (BD Biosciences) for 10 min; blood leukocytes were washed twice with DPBS. In addition, RCs (5 x 10^5^) were treated with 20 μg/mL of human IgG for 15 min, then surface-stained for CTLs and NK cells using the same protocol and reagents as those used for PB.

Intracellular flow cytometry was used in functional assays for CTLs and NK cells. After staining with extracellular markers (CTLs: CD3-APC-Cy7, CD4-APC, CD8-Pacific blue; and NK cells: anti-CD3-Pacific blue, CD16-FITC or APC, CD56-APC, CD45 PECy7), cells were permeabilized and fixed using anti-human FoxP3 staining set (eBioscience). The following antibodies were then added for CTLs: anti-IFN-γ-PECy7, IL-2-FITC, TNF-α-PerCP-Cy5.5, MIP-1β-PE, granzyme B-FITC and Perforin-PE; and for NK cells: anti-granzyme B-FITC, Perforin-PE, TNF-α-PerCP-Cy5.5 and anti-IFN-γ-APC-Cy7. The cells were incubated for 30 min at 4°C, washed twice with DPBS and fixed with 2% paraformaldehyde. At least 200.000 events were acquired in a FACS CANTO-II (BD Biosciences) and analyzed using the FlowJo Software version 9.7.5 (Tree Star, Inc, Ashland, OR, USA) or the FACSDiva 6.1.2 version (BD Biosciences). Appropriate isotype-matched control antibodies were included to define positive thresholds. Using side (SSC) vs. forward (FSC) light scatter we selected cells with compatible characteristics of viable cells. Then, the gate of lymphocytes, was used to analyze the following cell populations of T-cells: CD3^+^/CD4^+^ or CD3^+^/CD8^+^; and NK cells: CD3^-^/CD16^+^/CD56^dim^; CD3^-^/CD16^-^/CD56^dim^; CD3^-^/CD16^-^/CD56^bright^; CD3^-^/CD16^+^/CD56^-^); NK cells in GALT were reported as total NK cells (CD45^+^/CD3^-^/CD16^+^/CD56^+^). The gate strategy for the selection of T and NK cells, and for the analysis of activation markers are provided in the **S1 Fig.**

### Functional assays

#### NK cells

In 24-well tissue culture plates (Costar, Corning Inc., NY, USA), 1×10^6^ PBMCs/mL were stimulated with 50 ng/mL of a combination of IL-12 and IL-15 (BD Biosciences) and cultured for 48 hours at 37°C with 5% CO_2_. Monensin (1 mg/mL) (eBioscience) and Brefeldin A (1 mg/mL) (eBioscience) were added to the culture 24 hours before the end of the incubation time. Unstimulated cells were cultured in parallel. After incubation, the cells were washed with DPBS and stained for surface receptors, fixed, permeabilized and stained for intracellular molecules. The expression of molecules related to NK-cell function was measured in the total NK-cell population (CD3^-^/CD16^+^/CD56^+^). The gate strategy for the selection of responding cells are provided in the **S2 Fig**.

#### CTLs

After isolation of PBMCs or RCs, costimulatory antibodies anti-CD28 and anti-CD49 were added at a final concentration of 1 mg/mL (BD Biosciences), as well as Monensin (1 mg/mL) (eBioscience), Brefeldin A (1 mg/mL) (eBioscience) and anti-CD107a. The cells were then stimulated with HIV-1 Consensus B Gag-peptide pool (138 peptides) (NIH, Germantown, MD, USA). Peptide pools of Staphylococcal enterotoxin B (SEB) were used as positive control. Both, HIV-1 and SEB peptides were used at a final concentration of 10 μg/mL. Cells cultured with anti-CD28 and anti-CD49d served as background. PBMCs and RCs were incubated at 37°C, in 5% CO_2_ for 12 hours, stained for surface receptors, fixed, permeabilized and then stained for intracellular molecules.

Boolean gate analysis was applied for polyfunctional evaluation. Data are reported after background correction, and for mono and dual function subsets a level of 0.05% (after background subtraction) was considered as the threshold for a positive response. For three or more responses, the threshold was defined as 0.005% (after background subtraction). Data were analyzed using the SPICE software (version 5.35; NIH, Bethesda, MD). CD8^+^ T-cells responding in any way to Gag stimulation were used to analyze the total response. The gate strategy for the selection of responding cells are provided in the **S3 Fig**.

### Plasma LPS levels

The assay was performed according to the manufacturer’s instructions by the endpoint chromogenic LAL assay QCL-1000 (Lonza Inc, Allendale, NJ, USA) (sensitivity range 0.1–1.0 EU/mL). The background attributable to the turbidity of the diluted plasma was subtracted.

### Statistical analysis

The results are presented as median and range. To compare data from HIV controllers vs. HIV-progressors, a non-parametric test (Mann-Whitney *U—*two-tailed test) was performed. Correlation analyses were based on Spearman correlation coefficient calculations and the best-fit line was graphed using Linear regression. A *p* value <0.05 was considered statistically significant. To evaluate statistical differences of allelic frequencies of HLA, a Fisher exact test was used. The statistical tests were performed using the Graph-Pad Software version 5.00.

## Results

### CD4^+^ T-cells and some subpopulations of NK cells are increased in PB and GALT from HIV controllers

Although there were no differences in the time length after HIV diagnosis between both infected groups, as expected, HIV controllers exhibited significantly lower VL and higher CD4^+^ T-cell count in PB (**Table 1**).

**Fig 1:**
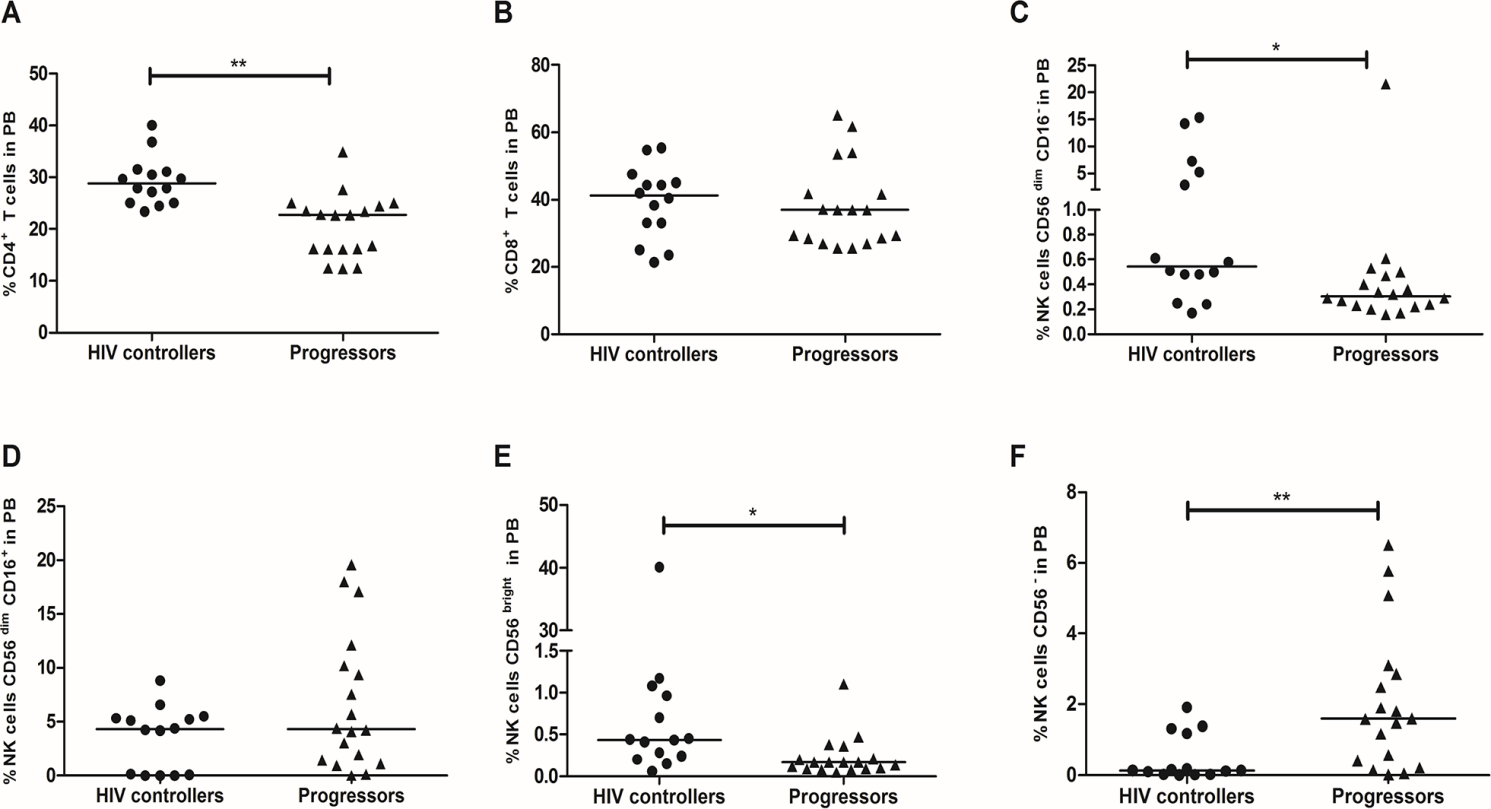
Frequency of T and NK cells in peripheral blood from HIV controllers and progressors. Peripheral blood was incubated with monoclonal antibodies against human molecules on (A) CD4^+^ T-cells. (B) CD8^+^ T-cells. (C) CD56^dim^/CD16^-^. (D) CD56^dim^/CD16^+^. (E) CD56^bright^. (F) CD56^−^, and detected by flow cytometry as described in Materials and Methods. The black line represents the median. A Mann Whitney test was used with a confidence level of 95%. Significant differences are indicated at the top of the figure. (*p<0.05, **p<0.01).

**Fig 2:**
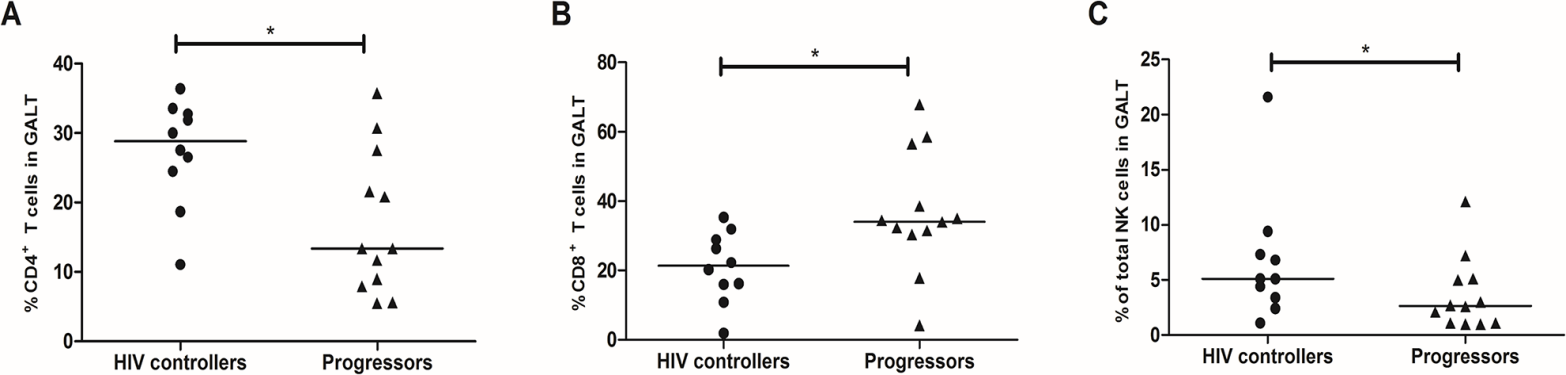
Frequency of T and NK cells in GALT from HIV controllers and progressors. Rectal cells were incubated with monoclonal antibodies against human molecules on (A) CD4^+^ T-cells. (B) CD8^+^ T-cells. (C) Total NK cells, and detected by flow cytometry as described in Materials and Methods. The black line represents the median. A Mann Whitney test was used with a confidence level of 95%. Significant differences are indicated at the top of the figure. (*p<0.05, **p<0.01).

The percentage of CD4^+^ T-cells was higher in PB and GALT from HIV controllers than progressors (29.7%, 23.4–39.9%; vs. 22.73%, 12.44–34.84%; *p* = 0.002 and 28.1%, 11.07–36.4%; vs. 13.3%, 5.5–35.8%; *p* = 0.039; **Figs 1A and 2A**). In addition, the frequency of CD8^+^ T-cells in PB was similar in both groups (41.1%, 21.4–55.4%; vs. 37.1%, 25.6–65.1%; *p* = 0.829; **Fig 1B**), but significantly lower in GALT from HIV controllers (21.3%, 1.2–35.4%; vs. 34.2%, 4.13–67.9%; *p* = 0.046; **Fig 2B**).

Furthermore, the percentage of NK CD56^dim^/CD16^-^ and NK CD56^bright^ were higher in PB from HIV controllers (0.6%, 0.2–15.3%; vs. 0.3%, 1.2–21.6%; *p* = 0.017 and 0.4%, 0.1–40.1%; vs. 0.2%, 0.05–1.1%; *p* = 0.011, respectively **Fig 1C and 1E**). No differences were observed in NK CD56^dim^/CD16^+^ (4.2%, 0–8.8%; vs. 4.3%, 0.02–19.6%; *p* = 0.12; **Fig 1D**). In contrast, NK CD56^−^ were significantly lower in HIV controllers (0.12%, 0–1.9%; vs. 1.6%, 0.02–6.5%; *p* = 0.008; **Fig 1F**). Finally, the frequency of total NK cells in GALT was increased in HIV controllers (5.1%, 1.1–21.6%; vs. 2.6%, 0.8–12.1%; *p* = 0.047; **Fig 2C**). A negative correlation was obtained between VL and percentage of NK CD56^bright^ (r = -0.5649; *p* = 0.0011, **S4 Fig**), while the percentage of NK CD56^−^ was positively correlated with VL (r = 0.4306; *p* = 0.0156, **S4 Fig**).

### Lower expression of activation molecules on CD4^+^ T and NK cells from HIV controllers

**Fig 3:**
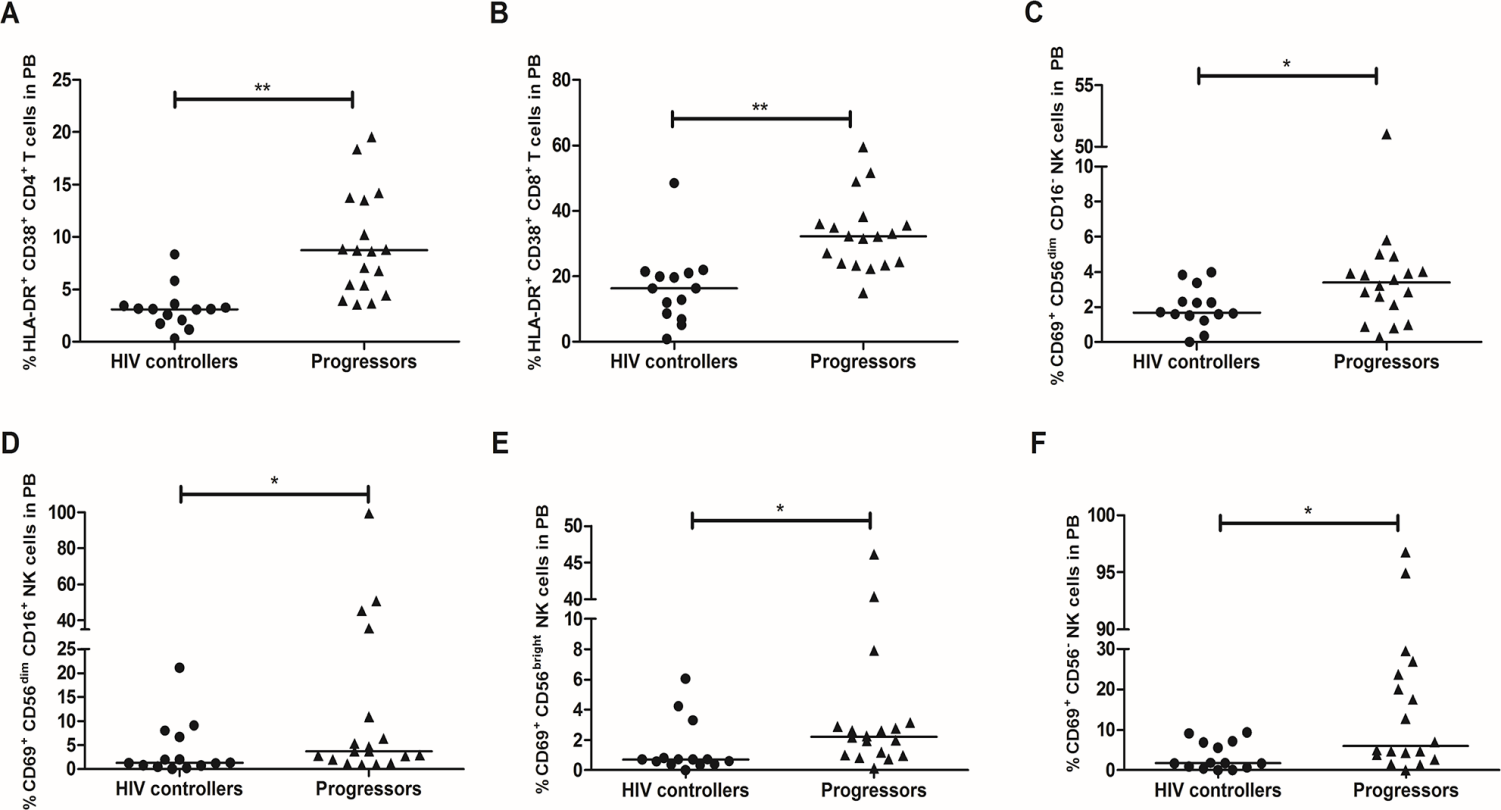
Expression of activation markers on T and NK cells from PB. Peripheral blood was incubated with monoclonal antibodies against HLA-DR and CD38 on T-cells: (A) CD4^+^ T-cells. (B) CD8^+^ T-cells; and CD69 on NK cells: (C) CD56^dim^/CD16^-^. (D) CD56^dim^/CD16^+^. (E) CD56^bright^. (F) CD56^−^, detected by flow cytometry as described in Materials and Methods. The black line represents the median. A Mann Whitney test was used with a confidence level of 95%. Significant differences are indicated at the top of the figure. (*p<0.05, **p<0.01).

**Fig 4:**
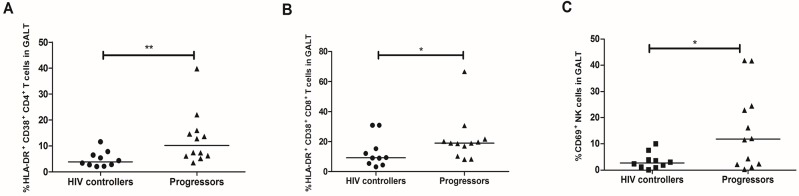
Expression of activation markers on T and NK cells from GALT. Rectal cells were incubated with monoclonal antibodies against HLA-DR and CD38 on T-cells: (A) CD4^+^ T-cells. (B) CD8^+^ T-cells; and (C) CD69 on total NK cells, detected by flow cytometry as described in Materials and Methods. The black line represents the median. A Mann Whitney test was used with a confidence level of 95%. Significant differences are indicated at the top of the figure. (*p<0.05, **p<0.01).

HIV controllers exhibited decreased co-expression of HLA-DR and CD38 on CD4^+^ T-cells from PB (3.1%, 0.3–8.3; vs. 8.7%, 3.6–19.5%; *p* = 0.0005; **Fig 3A**) and GALT (3.8%, 2.03–11.5%; vs. 10.1%, 3.5–39.8%; *p* = 0.005; **Fig 4A**), and also in CD8^+^ T-cells from PB (16.3%, 0.9–48.5%; vs. 32.2, 14.9–59.5%; *p* = 0.002; **Fig 3B**) and GALT (9.2%, 3.2–30.9%; vs. 18.9%, 8.3–66.6%; *p* = 0.045; **Fig 4B**).

In addition, HIV controllers exhibited lower expression of CD69 in the following NK cells from PB: NK CD56^dim^/CD16^-^ (1.7%, 0–3.9%; vs. 3.4%, 0.3%-51%; *p* = 0.047; **Fig 3C**); NK CD56^dim^/CD16^+^ (1.3%, 0–21.1%; vs. 3.7%, 0.9–99.5%; *p* = 0.045; **Fig 3D**); NK CD56^bright^ (0.72%, 0–6.1%; vs. 2.2%, 0.14–46.15%; *p* = 0.030; **Fig 3E**); and NK CD56^−^ (1.7%, 0–9.4%; vs. 6%, 0.15–96.7%; *p* = 0.045; **Fig 3F**). A significant positive correlation was observed between the percentage of cells NK CD56^bright^ CD69^+^ and the VL (r = 0.4306; *p* = 0.0156, **S4 Fig**).

Similar observations were obtained in GALT, where the percentage of total NK cells expressing CD69 was lower in HIV controllers than in HIV-progressors (2.7%, 0.1–10%; vs. 12%, 0.3–41.8%; *p* = 0.04; **Fig 4C**).

#### Lower levels of plasma LPS in HIV controllers

**Fig 5:**
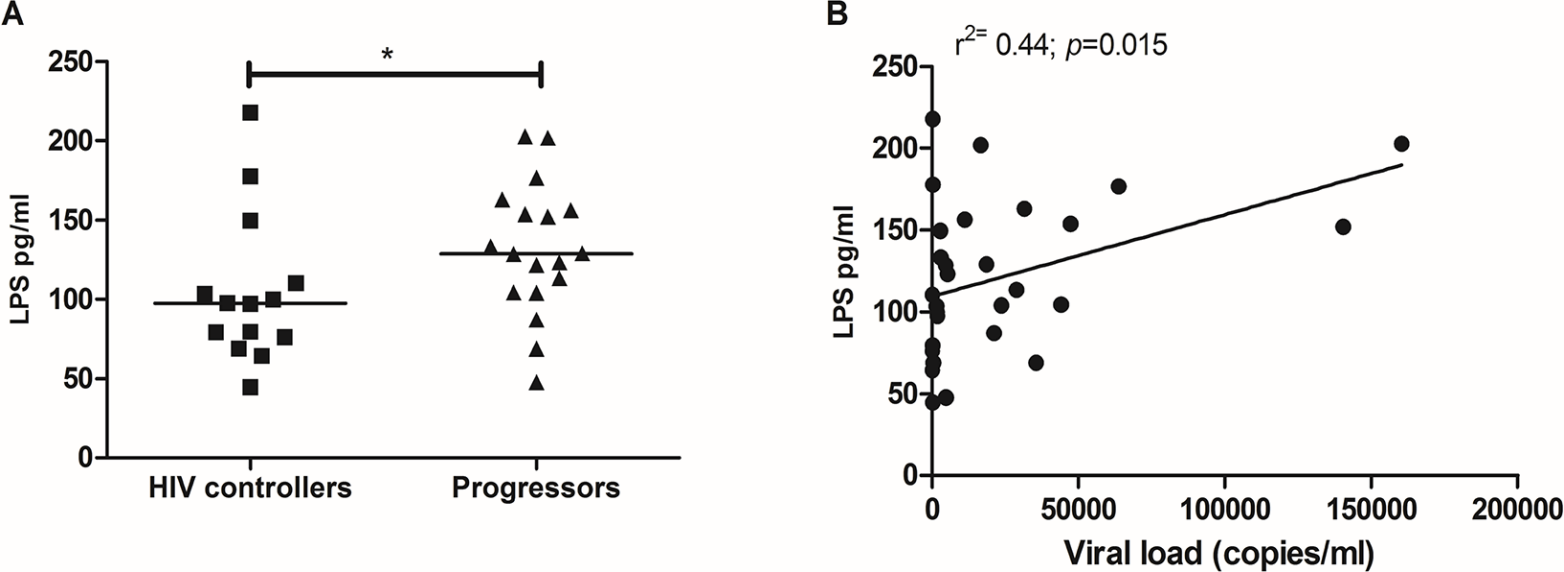
Plasma LPS levels. (A) Comparison of the plasma levels in pg/mL of LPS in HIV controllers and progressors measured by the LAL assay and (B). The black line represents the median. A Mann Whitney test was used with a confidence level of 95%. Significant differences are indicated at the top of the figure. (*p<0.05).

As plasma levels of LPS have been strongly associated with immune activation and disease progression, we measured this parameter in both cohorts. HIV controllers have significantly lower levels of plasma LPS compared to progressors (97.6 pg/mL (44.8 pg/mL– 218 pg/mL); vs. 128.9 pg/mL (47.7 pg/mL– 202.7 pg/mL respectively); *p* = 0.04; **Fig 5A**). In fact, a positive correlation between LPS levels and viral load was observed when all infected patients were included in the analyses (Spearman r = −0.44; *p* = 0.015, **Fig 5B**).

### CTLs from HIV controllers exhibit higher function in response to HIV Gag peptides

**Fig 6:**
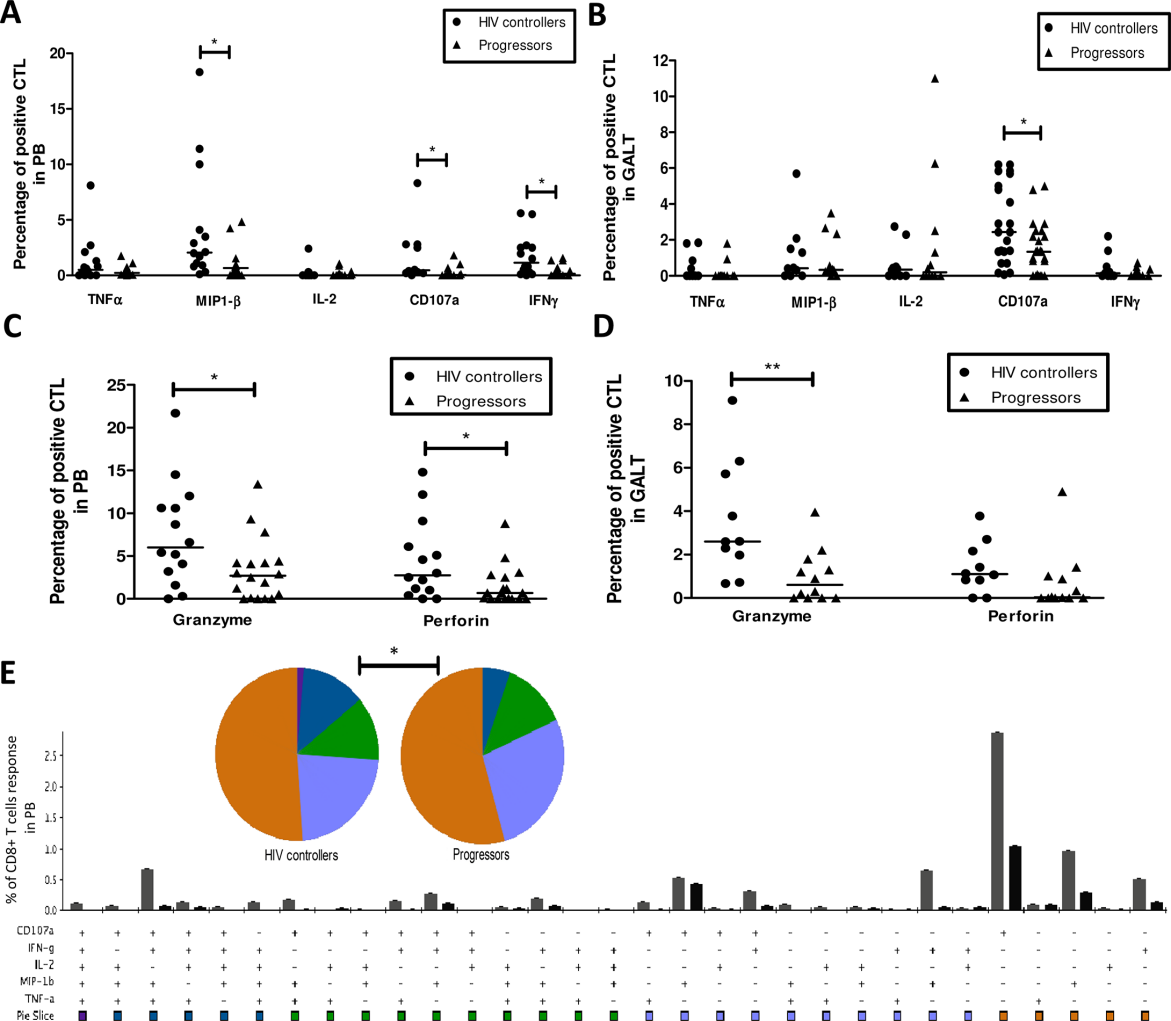
Activity of CTLs from peripheral blood and GALT of HIV controllers and progressors. (A-D) Peripheral blood and rectal cells were stimulated with HIV-1 Consensus B Gag-peptide pool during 12 h, and then monoclonal antibodies against TNFα, MIP-1β, IL-2, CD107a, IFN-γ, granzyme B and perforin, were added. The expression of these molecules was detected by flow cytometry as described in Materials and Methods. The results are presented as median, range minimum and maximum. (E) Comparison of the polyfunctional profiles of Gag-specific responses in PB. The slices of the pies correspond to the proportions of Gag-specific CD8^+^ T-cells (as a frequency of the total Gag response) expressing 1, 2, 3, 4, or 5 functions, calculated using Boolean gating. A Mann Whitney test was used with a confidence level of 95%. Significant differences are indicated at the top of the figure. (*p<0.05, **p<0.01).

HIV controllers exhibited a higher percentage of PB CD8^+^ T-cells expressing MIP-1β (2%, 0.1–18.3%; vs. 0.66%, 0–4.8%; *p* = 0.032); CD107a (0.45%, 0–8.3%; vs. 0.04%, 0–1.8%; *p* = 0.012), and IFN-γ (0.8%, 0–5.6%; vs. 0.15%, 0–1.6%; *p* = 0.037). In contrast, the percentage of cells expressing TNF-α (0.4%, 0–8.1%; vs. 0.21%, 0–1.8%; *p* = 0.602) and IL-2 (0%, 0–2.4%; vs. 0%, 0–1.05%; *p* = 0.069) was similar (**Fig 6A**).

In GALT we found a higher release of CD107a in HIV controllers (2.4%, 0.07–6.2%; vs. 1.3%, 0–5%; *p* = 0.022). However, no differences were observed in the production of cytokines: TNF-α (0.01%, 0–1.84%; vs. 0%, 0–0.94%; *p* = 0.08); MIP-1β (0.43%, 0–5.7%; vs. 0.46%, 0–4.1%; *p* = 0.703); IL-2 (0.35%, 0–2.74%; vs. 0.3%, 0–11%; *p* = 0.72); and IFN-γ (0.15%, 0–2.2%; vs. 0%, 0–0.73%; **Fig 6B**).

In addition, in independent experiments we evaluated the expression of granzyme B and perforin in CD8^+^ T-cells stimulated with HIV peptides. In PB we found a significantly increased expression of granzyme B (6%, 0–21.7%; vs. 2.9%, 0–13.4%; *p* = 0.048) and perforin (2.75%, 0–14.8%; vs. 0.7%, 0–8.8%; *p* = 0.0319) in Gag-stimulated cells from HIV controllers; **Fig 6C**. In GALT the expression of granzyme B was higher in HIV controllers (2.6%, 0.7–9.1%; vs. 0.6%, 0–3.9%; *p* = 0.0042), while the difference in the production of perforin was not statistically significant (1.1%, 0–3.8%; vs. 0.03%, 0–4.9%; *p* = 0.35; **Fig 6D**).

Polyfunctional analysis were also performed to determine the complexity of the response. We observed that HIV controllers have a significantly higher percentage of polyfunctional CTLs responding with 4 functions (blue pie slice, in PBMCs (*p* = 0.0032; **Fig 6E**) but not in GALT (*p* = 0.456; **S5 Fig**). Although pie proportion distribution suggests a higher frequency of the 5-functions category in PBMCs in HIV controllers than in progressors, this difference was not statistically significant (*p* = 0.092).

### Higher activity of NK cells from HIV controllers

**Fig 7:**
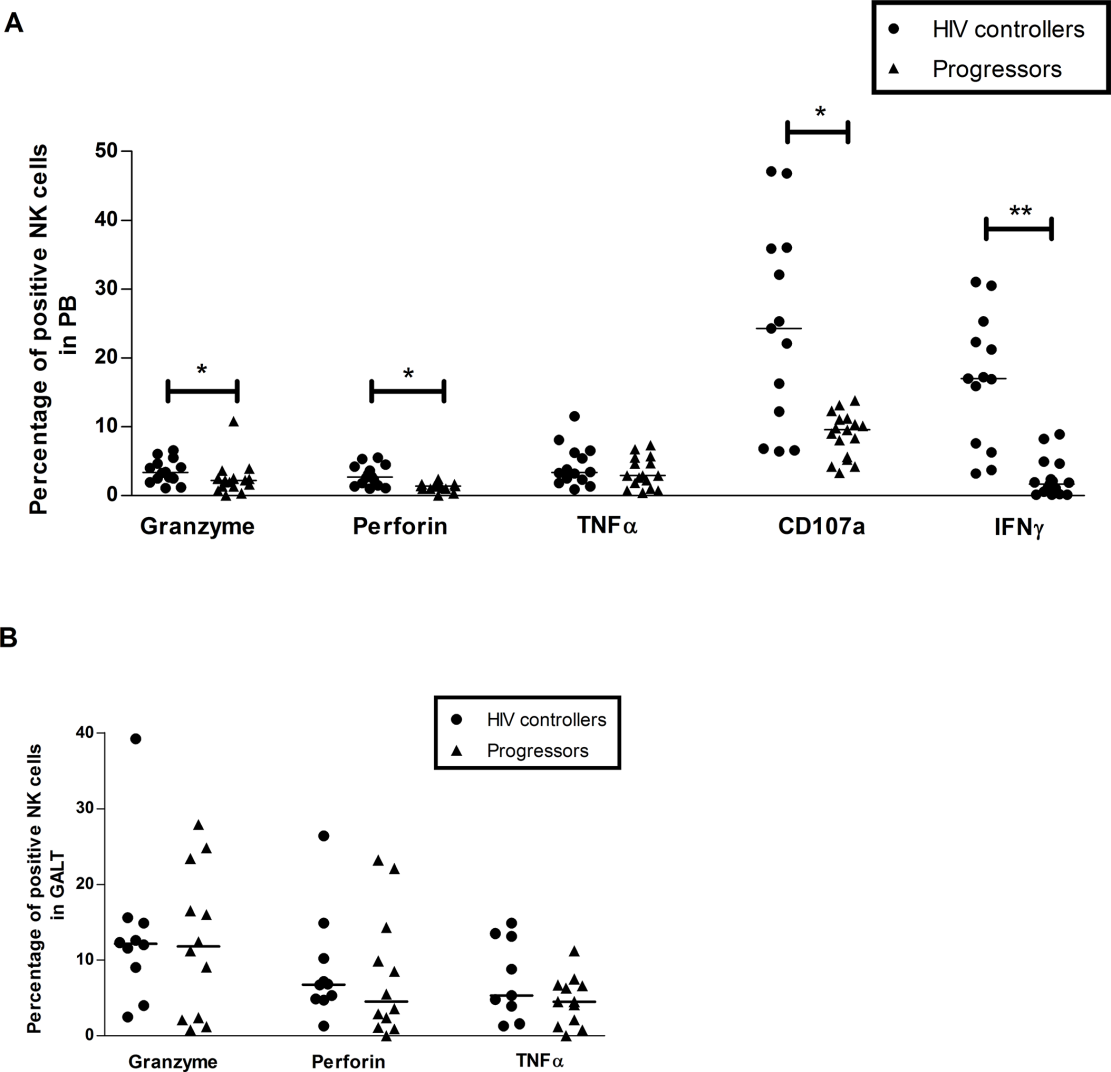
Activity of NK cells after stimulation with cytokines. Peripheral blood (A) and rectal cells (B) were stimulated with IL-12 and IL-15 during 48 h, and then monoclonal antibodies against granzyme B, perforin, TNFα, CD107a and IFN-γ were added. The expression of these molecules was detected by flow cytometry as described in Materials and Methods. The results are presented as median, range minimum and maximum. A Mann Whitney test was used with a confidence level of 95%. Significant differences are indicated at the top of the figure. (*p<0.05, **p<0.01).

The activity of NK cells from PB of HIV controllers was stronger, as the percentage of NK cells expressing granzyme B (3.4%, 1.1–6.6%; vs. 2.2%, 0–10.8%; *p* = 0.039); perforin (2.7%, 1–5.5%; vs. 1.4%, 0–2.4%; *p* = 0.04); CD107a (24.2%, 6.4–47.1%; vs. 9.6%, 3.3–13.8%; *p* = 0.032); and IFN-γ (16.9%, 3.2–31%; vs. 1.6%, 0.1–8.9%; *p* = 0.002), was increased. The percentage of cells expressing TNF-α was similar in both groups (3.4%, 0.9–11.5%; vs. 2.9%, 0.4–7.3%; *p* = 0.339), **Fig 7A**.

In contrast, we observed that the activity of NK cells in GALT was comparable, as the percentage of cells expressing granzyme B (12%, 2.5–39.2%; vs. 11.8%, 0.8–27.9%; *p* = 0.859); perforin (6.7%, 1.3–26.4%; vs. 4.6%, 0–23.2%; *p* = 0.413); and TNF-α (5.3%, 1.3–14.9%; vs. 4.5%, 0–11.2%; *p* = 0.240) did not exhibit significant differences, **Fig 7B**. CD107a and IFN-γ were not measured in GALT due to sample limitations.

## Discussion

HIV controllers are a relevant model to explore mechanisms associated with viral control [6]. To determine the role of the cytotoxic response in the spontaneous viral control, we evaluated a cohort of HIV controllers who were compared with progressors. When we characterized T and NK cells from PB and GALT of HIV controllers, we found that HIV controllers exhibit higher frequency of CD4^+^ T-cells and some subpopulations of NK cells as well as lower immune activation and higher cell function compared to progressors.

Mucosal and peripheral depletion of CD4^+^ T-cells constitutes the most important marker of AIDS progression [17,18]. Our HIV controllers exhibited higher frequency of CD4^+^ T-cells in PB and lower levels of immune activation than progressors. Supporting our results, other authors reported conserved immune parameters in HIV controllers [19–22]. It is well known that one of the major contributors to chronic immune activation is the damage of mucosal barriers, resulting in increased microbial translocation, as evidenced by increased levels of plasma LPS [3]. We found decreased levels of LPS in plasma from HIV controllers compared with progressors and a positive correlation between LPS levels and VL was observed, suggesting that the control of HIV replication is associated with diminished levels of microbial translocation. In contrast to what was expected, LPS levels did not correlate with immune activation or with other immune parameters. Three outliers among HIV controllers were responsible for the unanticipated results. In these patients, the presence of additional infections may have influence the LPS levels; however, this requires further analysis.

HIV infection has a great impact on NK cells [23–25]. The most common alterations of NK cells during infection includes: decreased frequency of subsets with high activity, such as CD56^bright^ or CD56^dim^ [26]; expansion of dysfunctional CD56^-^ cells [26]; apoptosis [27]; and low cytotoxic activity and production of cytokines [27]. These cells are crucial during the antiviral immune response and seem to be involved in delaying AIDS progression, as suggested in LTNP, who exhibit high frequency of cytotoxic NK cells (CD56^dim^), strong functional activity, and high production of RANTES, MIP-1α and MIP-1β that are negatively correlated with VL [28]. Similarly, we found higher frequency of CD56^bright^ and/or CD56^dim^ NK cells, and lower percentage of dysfunctional CD56^-^ cells in HIV controllers. Although NK CD56^dim^ is the subset most strongly associated with cytotoxic activity, some studies reported that NK CD56^bright^ have also degranulation capacity, suggesting that NK cells have a high plasticity to respond during infections using several mechanisms [29]. It is important to note that in the HIV controllers group we found a wide range in the percentage of CD56^bright^ or CD56^dim^ CD16^-^ NK cells, as some of our patients exhibited a surprisingly high frequency of these subsets. Those observations were confirmed using different monoclonal antibodies targeting CD16 and suggest that at least in some of the HIV controllers, the CD56^bright^ and CD56^dim^ CD16^-^ NK cells might be related with the spontaneous control of viral replication. CD56^dim^ CD16^+^ and CD56^dim^ CD16^-^ NK cells share similar functional activity, but it is recognized that the first subpopulation exhibits the strongest cytotoxic capacity among NK cells subpopulation. However, it was recently shown that during HIV infection less-differentiated CD56^dim^ CD16^+^ NK cells are preferentially depleted, suggesting that the maintenance of high levels of CD56^dim^ CD16^-^ NK cells could be crucial for the anti-HIV response [30].

We also observed that all NK-cell subpopulations in HIV controllers exhibited lower levels of activation, which is in compliance with previous studies that associated disease progression and the level of viral replication with the expression of activation markers on NK cells [23,31]. In fact, NK cells can be activated trough the TLR pathway, as previously demonstrated using HIV-derived ssRNA, suggesting that the low level of expression of activation molecules in HIV controllers could be associated with the reduced number of viral particles [32]. Regarding NK cells in GALT, there are few studies evaluating their role in the preservation of rectal mucosa during HIV infection [12,32]. Some reports suggest that viral replication impacts on their frequency as observed in HAART-suppressor patients who exhibited increasing percentages of NK cells in colonic tissue after effective treatment [11]. We did not observe any differences in the frequency of total NK cells in GALT, but the expression of CD69 was decreased in HIV controllers, suggesting minor alterations of these cells in mucosal tissue. Although this work did not include a group of seronegative individuals, we have previously reported that HIV controllers have preserved immune parameters on blood and gastrointestinal mucosa, including similar frequency and phenotype of T and NK cells compared to uninfected individuals [33]. In addition, although gender may influence immune responses, similar distribution by gender was not possible. However, analysis of data restricted by gender were performed and the results were similar than those observed in the whole population.

HIV controllers exhibited increased production of molecules associated with the cytotoxic activity of CTLs in both tissues, and a higher percentage of polyfunctional cells responding with 4 functions in PB. The response mediated by CTLs has been previously associated with control of HIV infection and is considered a key mechanism associated with an effective antiviral immune response [7,8,13]. Some studies have reported interesting results regarding CTL activity in PB, including: i) long survival [34]; ii) efficient capacity to eliminate infected CD4^+^ T-cells [34]; iii) high production of effector molecules [35,36]; iv) early inhibition of viral replication [35]; v) broad variant reactivity [37]; and vi) high-avidity Gag-specific HLA-B-restricted responses [38], among others. Strong GALT CTL responses mediated mainly by the production of cytokines have been shown [39]. Although we did not observe differences between HIV controllers and progressors in the production of cytokines in GALT, a potent response mediated by the degranulation capacity (CD107a upregulation) and production of granzyme B in HIV controllers, was found. Interestingly, we also observed increased expression of CD107a, granzyme B and perforin on PB CTL, suggesting that the effector cytotoxic function might play a crucial role for viral control on the periphery and GALT, and could be involved in preserving the architecture of mucosal tissue.

Granzyme B is a cytolytic enzyme that mediates cellular apoptosis of infected cells [40], being a key cytotoxic mediator. In fact, senescent T-cells exhibit a reduced expression of granzyme B associated with low response [41]. When the mucosal and systemic poxvirus prime-boost vaccines were evaluated, a higher expression of granzyme B was induced in genitorectal lymphocytes, suggesting an important role of this protein in mucosal sites [42]. Supporting our results, previous studies in LTNP found that the high cytotoxic capacity to eliminate infected cells from these patients is mediated by granzyme B [43]. In contrast, progressor patients exhibit altered expression of this protein and deficient loading of lytic granules that is associated with the loss of viral control [43,44]. Our observations suggests that the activity mediated by granzyme B could be important to control viral replication in GALT, but further information on the level of viral replication in this tissue in HIV controllers would be required to clearly define the role of this enzyme in gut antiviral responses. Moreover, cells residing in this tissue are under excessive pressure, as this site concentrates the highest number of cells replicating HIV in the body, and the cytokine-mediated immune balance could be difficult to maintain. Instead, a strong capacity to eliminate infected cells could be more efficient and result in lower alterations of immune components in peripheral and mucosal tissues.

Based on the complexity of this viral—host interaction it is difficult to establish if the immune parameters described in HIV controllers are the cause or the consequence of the viral control. However, taking into account that innate responses, including NK effector mechanisms are crucial in the antiviral response, and based on our results, we are proposing a model in which during early events of the infection, innate molecules and cells may down regulate viral replication, resulting in less tissue damage and lower number of viral reservoirs. Then, in the chronic phase, adaptive immune responses including cytotoxic cells, exerts a potent viral control avoiding extensive immune alterations.

## Supporting Information

S1 Fig Gate strategy for the selection of T cells, NK cells and activation markers. (PDF)

S2 Fig Gate strategy for the selection of responding NK cells. (PDF)

S3 Fig Gate strategy for the selection of responding CD8+ T-cells (PDF)

S4 Fig Correlations of immune parameters and viral load (TIF)

S5 Fig Polyfunctional analysis of CTLs in GALT (PDF)
