# Supplementary figures and images for: Higher Frequency of NK and CD4+ T-Cells in Mucosa and Potent Cytotoxic Response in HIV Controllers
Source: PLoS One. 2015 Aug 20;10(8):e0136292. doi: 10.1371/journal.pone.0136292 (PMC4546229; doi:10.1371/journal.pone.0136292)

## A. Frequency and phenotype of T-cells

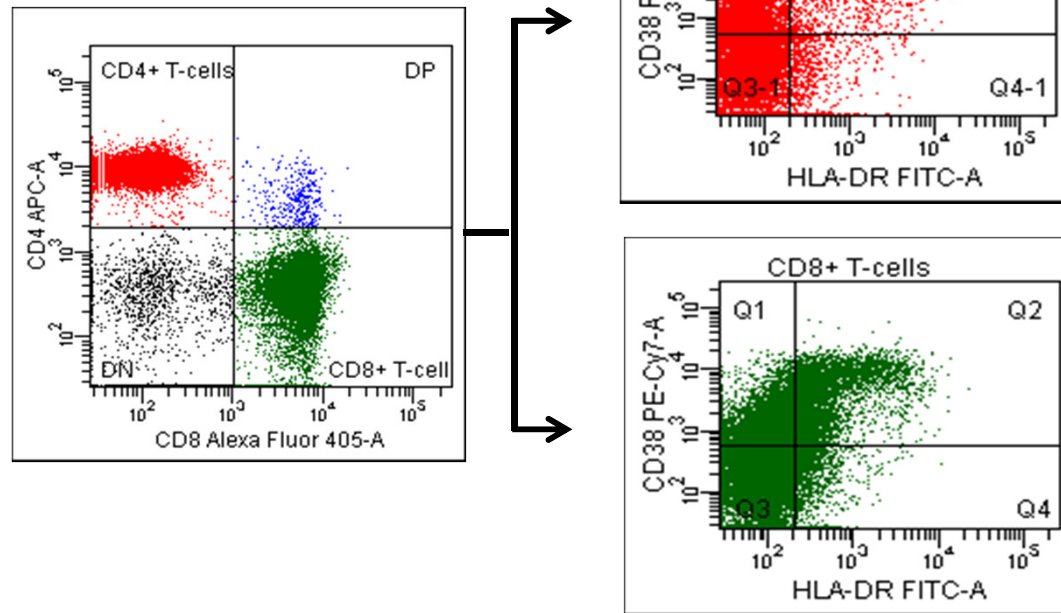

## B. Frequency and phenotype of NK cells

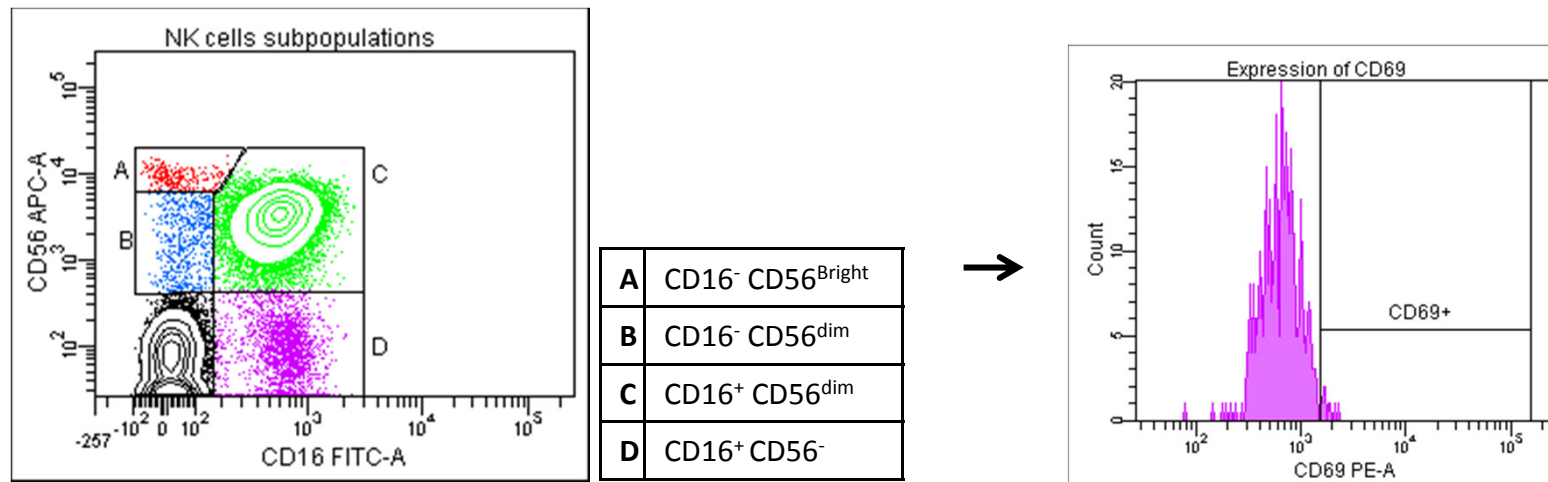

Supplement: S1 Fig — (PDF) [file pone.0136292.s001.pdf]

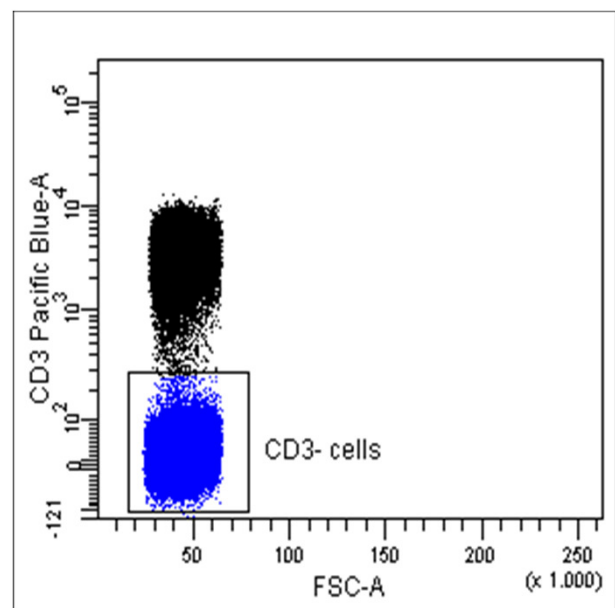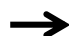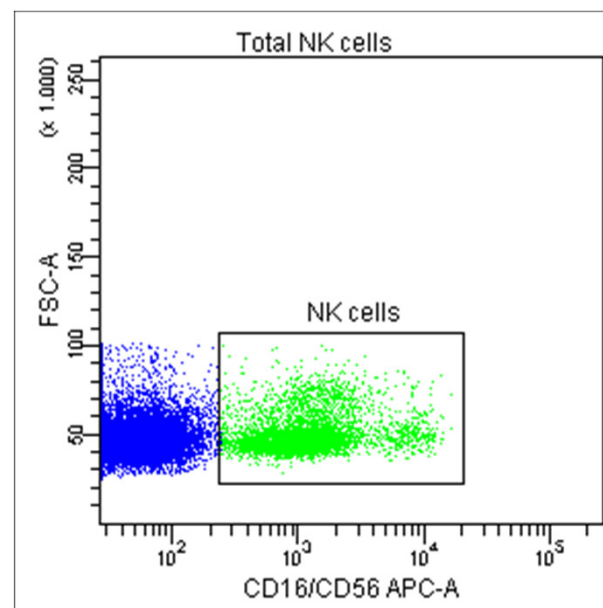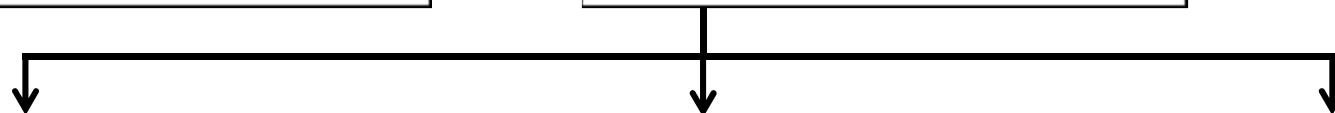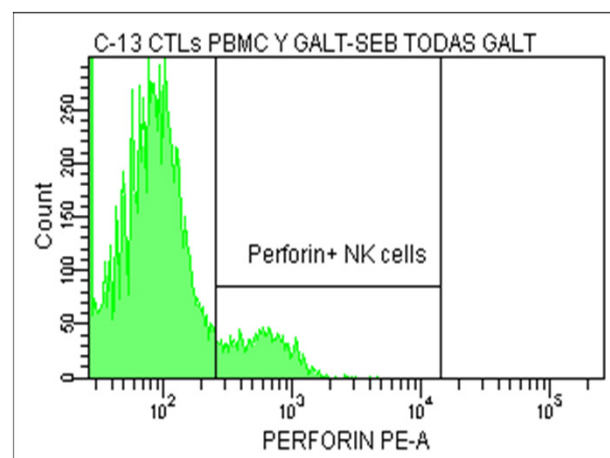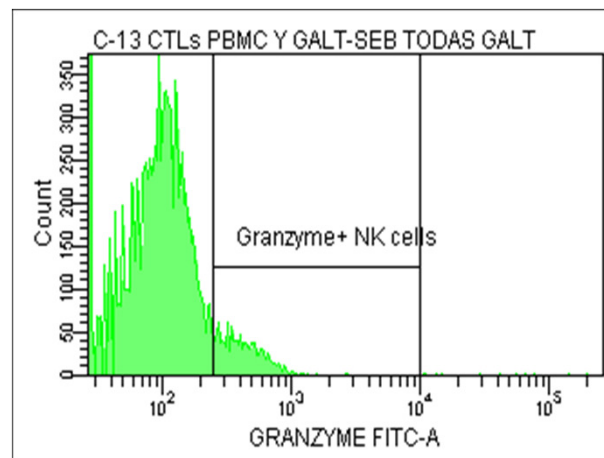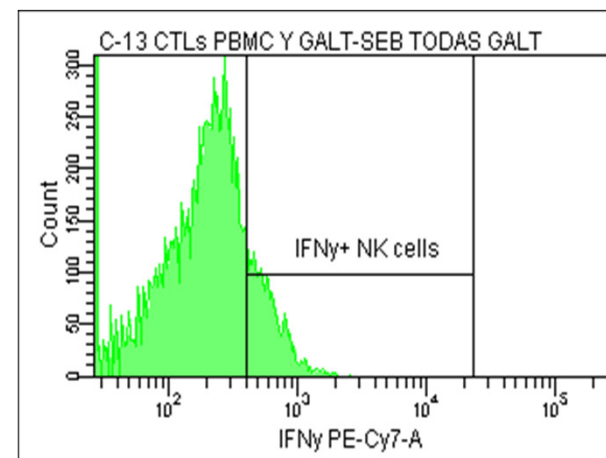

Supplement: S2 Fig — (PDF) [file pone.0136292.s002.pdf]

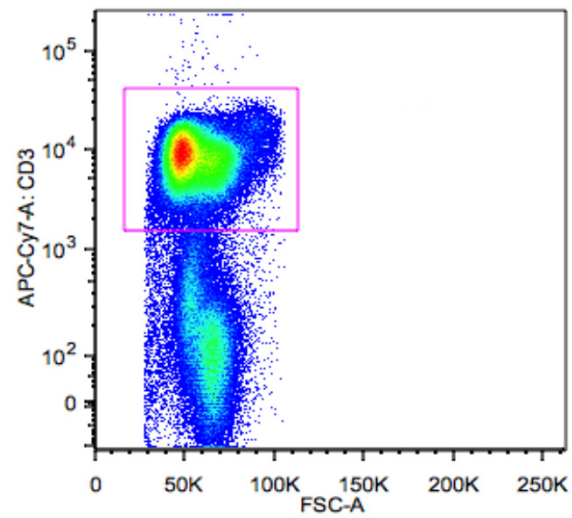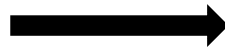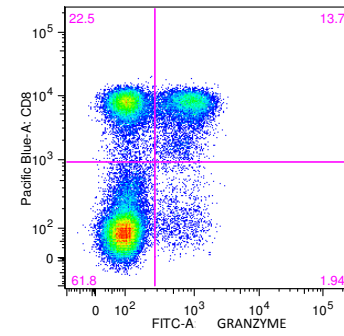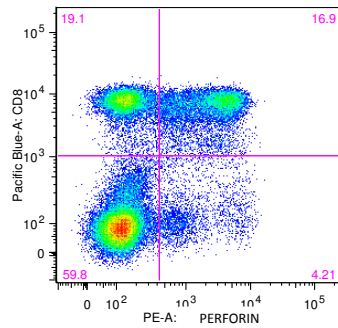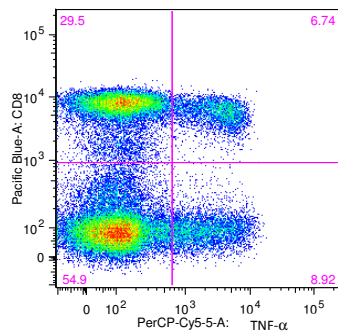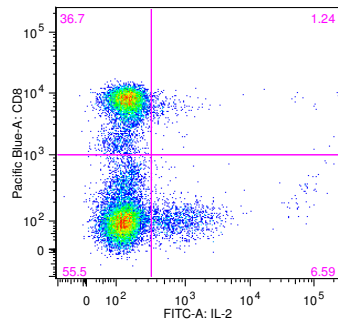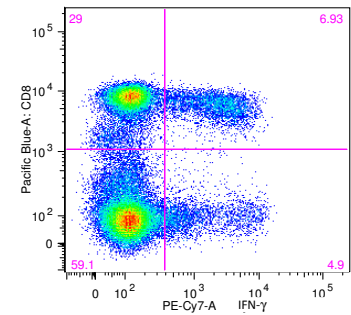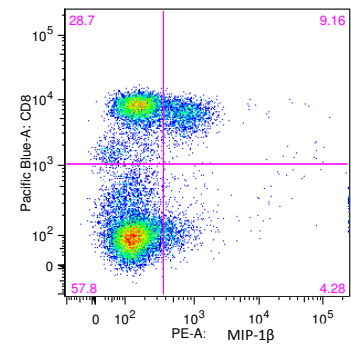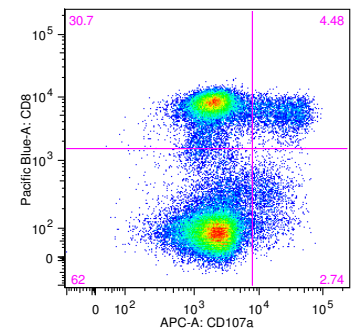

Supplement: S3 Fig — (PDF) [file pone.0136292.s003.pdf]

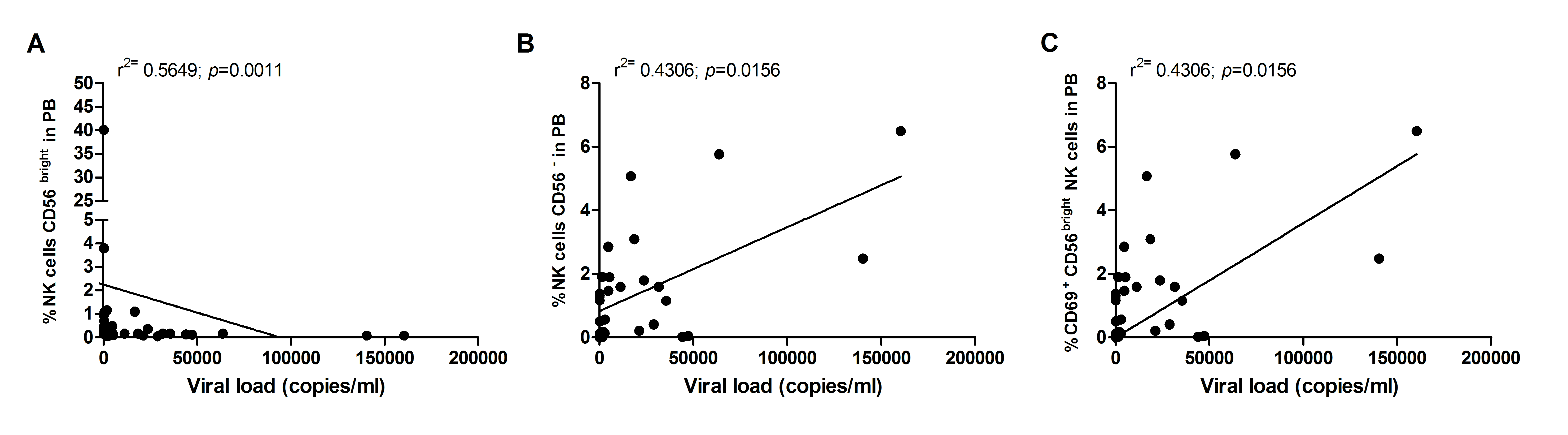

Supplement: S4 Fig — (TIF) [file pone.0136292.s004.tif]

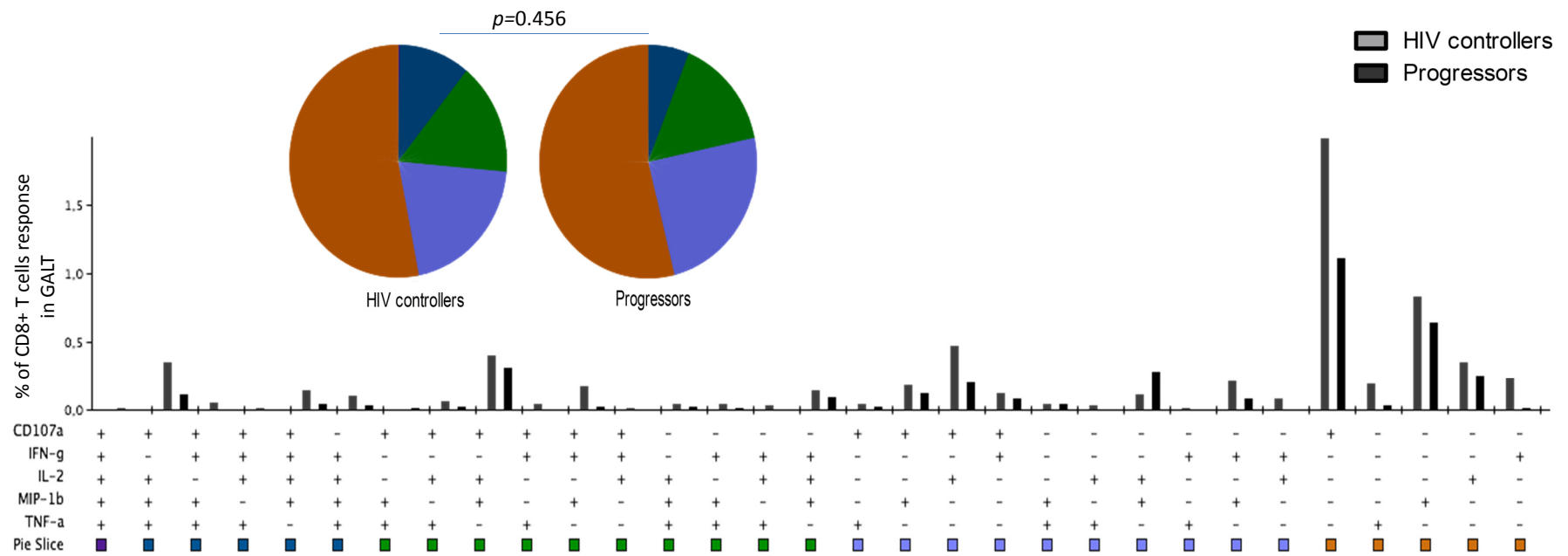

Supplement: S5 Fig — (PDF) [file pone.0136292.s005.pdf]
